# Eat, sleep, repeat: the role of the circadian system in balancing sleep–wake control with metabolic need

**DOI:** 10.1016/j.cophys.2020.02.003

**Published:** 2020-06

**Authors:** Rebecca C Northeast, Vladyslav V Vyazovskiy, David A Bechtold

**Affiliations:** 1 Centre for Biological Timing, Faculty of Biology, Medicine and Health, University of Manchester, Manchester, M13 9PT, UK; 2 Department of Physiology, Anatomy and Genetics, University of Oxford, Parks Road, Oxford, OX1 3PT, UK

## Abstract

Feeding and sleep are behaviours fundamental to survival, and as such are subject to powerful homeostatic control. Of course, these are mutually exclusive behaviours, and therefore require coordinated temporal organisation to ensure that both energy demands and sleep need are met. Under optimal conditions, foraging/feeding and sleep can be simply partitioned to appropriate phases of the circadian cycle so that they are in suitable alignment with the external environment. However, under conditions of negative energy balance, increased foraging activity must be balanced against sleep requirements and energy conservation. In mammals and many other species, neural circuits that regulate sleep and energy balance are intimately and reciprocally linked. Here, we examine this circuitry, discuss how homeostatic regulation and temporal patterning of sleep are modulated by altered food availability, and describe the role of circadian system in adaptation to metabolic stress.

**Current Opinion in Physiology** 2020, **15**:183–191

This review comes from a themed issue on **Physiology of sleep**

Edited by **Vladyslav Vyazovskiy** and **Jenny Morton**

For a complete overview see the Issue and the Editorial

Available online 28th February 2020

**https://doi.org/10.1016/j.cophys.2020.02.003**

2468-8673/© 2020 The Authors. Published by Elsevier Ltd. This is an open access article under the CC BY license (http://creativecommons.org/licenses/by/4.0/).

## Introduction

The majority of animals display consolidated patterns of behaviour across the 24 hour day, driven both by fluctuations in the external environment and by robust internal circadian clock timing. These behaviours are commonly partitioned into specific phases of the circadian cycle, across which sleep–wake and feed-fast cycles are segregated. Nevertheless, across the animal kingdom, there exists huge variation in the amount and temporal arrangement of wakefulness and sleep. For example, herbivorous species, such as ungulates, must dedicate large proportions of time to feeding, with sleep being drastically decreased. In contrast, large carnivorous species may have sporadic, yet energy rich meals, with the remaining time often dedicated to sleep. However, even within an individual organism, acute or chronic changes in environmental conditions, including food availability and associated energy state reveal profound flexibility in feeding and sleep–wake behaviour [1]. Food scarcity represents a major metabolic challenge whereby arousal promoting pathways enhance and increase food seeking behaviours. This altered behavioural priority must be balanced with strategies for energy conservation and with the need to satisfy essential sleep requirements. During periods of metabolic stress, a remarkable flexibility in sleep behaviour is evident across diverse species, where sleep timing, duration and architecture can be altered to accommodate foraging behaviours or to preserve energy. For example, from insects to mammals, sleep duration is typically decreased during acute short-term fasting [1, 2, 3], while during periods of prolonged starvation in some species, such as *Caenorhabditis elegans* and *Astyanax mexicanus* (blind Mexican cave fish), sleep can be substantially increased as a means of energy conservation [4,5]. Similarly, *Drosophila* selectively bred for starvation resistance demonstrate drastically increased sleep during extended periods of fasting [6], while sleep deprivation in flies results in reduced metabolic rates during recovery [7]. These examples highlight the responsiveness of sleep–wake control to metabolic challenges, and highlight the existence of a fundamental relationship between sleep and metabolism, as well as inherent flexibility within sleep regulatory mechanisms, which ultimately allows to maximise survival.

The circadian timing system contributes to both sleep regulation and energy homeostasis. In mammals, a master circadian clock in the suprachiasmatic nuclei (SCN) directs the overall temporal organisation of behaviour and physiology, including the sleep and wake cycle, feeding behaviour, body temperature and energy expenditure [8]. Through the activity of the SCN and its input and output pathways, temporal information is integrated to control both sleep and energy balance. In rats, destruction of the SCN eliminates temporal consolidation of sleep over 24 hour, while overall amount of sleep is maintained [9], thus demonstrating its role in the organisation of sleep but suggesting a limited role in sleep homeostasis. While the SCN itself is relatively insensitive to altered metabolic state (i.e. fasting duration or meal timing), the circadian system as a whole is adaptable to changes in food availability. As we discuss below, the circadian system plays a central role in the re-organisation of sleep, foraging and energy conservation mechanisms under conditions of metabolic stress.

## Neural correlates of sleep and its integration with feeding and circadian processes

**Figure 1:**
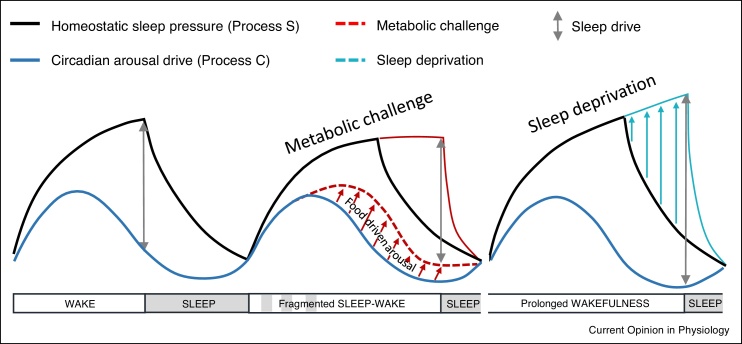
Adapted two-process model of sleep regulation. The black line represents the homeostatic process S, and the blue line represents process C of circadian arousal. The highest level of sleep drive is when process S is high and process C is low. During metabolic challenges such as restricted feeding (red line), there is a reduced sleep drive accrued through prolonged wakefulness, postulated here through a decrease in process S during food driven arousal. Conversely, following standard sleep deprivation, process S increases while the circadian drive process C remains unchanged, thus leading to a higher sleep drive. This model suggests ways in which sleep drive is not increased during times of metabolic need to allow behavioural flexibility, enabling animals to maintain arousal to seek food.

A longstanding framework for sleep regulation is the two-process model (Figure 1) based on the interaction of homeostatic (S) processes and circadian (C) processes. Homeostatic drives reflect wake-state-dependent and sleep-state-dependent accumulation and dissipation of sleep pressure (respectively), while circadian processes shape the temporal organisation of sleep and arousal state [10]. Our current understanding of the homeostatic arm is that it works much like an hourglass timer, wherein time spent in the waking state leads to an accumulation of sleep debt which must be dissipated during sleep [11]. Although specific substrates underlying homeostatic sleep drive remain elusive, a number of factors have been proposed, including increased adenosine levels in the basal forebrain [12], progressive changes in neuronal plasticity or excitability [13], and the need to maintain cellular homeostasis [14]. The circadian process maintains arousal to counteract the build-up of sleep pressure during the waking phase of the daily cycle, and then supports a sleep permissive state at an appropriate time, allowing temporal consolidation of sleep [15].

**Figure 2:**
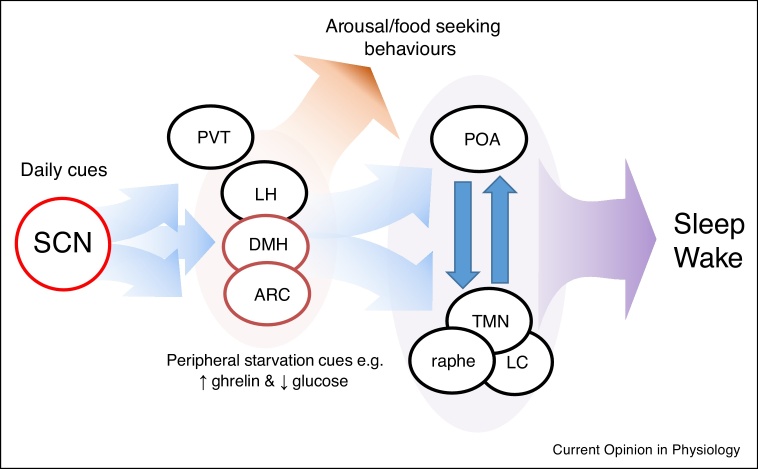
Neural circuits involved in circadian and metabolic regulation of arousal and sleep. The SCN (a master oscillator, red outline) is phase set by external timing cues, primarily light/dark signals, and directly connects to hypothalamic feeding centres the Arc and DMH (robust semi-autonomous oscillators, dark red outline) via primarily GABAergic connections to inhibit arousal during the day. The DMH and LH integrate peripheral metabolic signals to either inhibit sleep promoting regions in the POA or activate arousal and food seeking behaviours. The SCN also directly interacts with the PVT, an area also heavily implicated in modulating arousal response to metabolic need. ARC = arcuate nucleus, DMH = dorsomedial hypothalamus, LH = lateral hypothalamus, POA = preoptic nucleus, PVT = paraventricular thalamus, SCN = suprachiasmatic nuclei, TMN = tuberomamillary nucleus.

In its simplest form, the neural architecture of sleep involves a balanced and reciprocal inhibition between typically sleep active (e.g. ventrolateral [VLPO] and median preoptic area [POA]) and arousal promoting (e.g. locus coeruleus [LC] and tuberomammillary nucleus [TMN]) regions. The dominance of either side, and thus sleep–wake state, is determined by homeostatic, circadian and arousal systems. Of course, fine control of sleep is very complex with involvement of many areas and sleep-modifying factors contributing to sleep dynamics [16]. It is now eminently clear that multiple bidirectional connections exist between sleep and feeding control centres [17,18] (Figure 2). Nuclei within the hypothalamus and brainstem integrate sensory, endocrine and nutrient signals related to feeding and energy state in order to direct appropriate behavioural and physiological responses. These responses include the modulation of arousal and sleep processes. In particular, the lateral hypothalamus (LH) and mediobasal hypothalamus (MBH) contain a number of regulatory nuclei which serve to integrate energy balance with sleep state.

The LH contains orexin neurons which project extensively throughout the brain and form an important component of the sleep/wake controlling network [19, 20, 21, 22]. Orexin acts to promote wakefulness by increasing the activity of arousal circuits, such as the LC and TMN [23], and loss of orexin function leads to instability in sleep onset and poor sleep–wake maintenance [24]. Importantly, the orexin system plays an important role in mediating arousal in response to food scarcity and negative energy balance, acutely inhibiting sleep to satisfy immediate caloric requirements [19,20]. The electrical activity of orexin neurons is modulated by energy state in direct response to hormone and nutrient signals, and via shared anatomical innervation by feeding centres such as the arcuate nucleus (arc) and dorsomedial hypothalamus (DMH) [21]. Orexinergic signalling drives increased wakefulness during acute fasting [25], and intracerebroventricular (icv) injection of orexin in rodents can elicit feeding behaviour during normal sleep periods [25]. Orexin has also has been shown to inhibit SCN neuronal activity [26], and thus orexinergic feedback to the central clock may serve to directly modulate SCN control should arousing metabolic stimuli require temporal flexibility. The LH also contains a population of melanin concentrating hormone (MCH) neurons, which have been shown to influence sleep structure (in particular the duration and timing of REM sleep) in response to metabolic hormones [27^•^].

The DMH is another established hub responsible for the integration of sleep and metabolic processes [28]. Neurons in the DMH respond directly to changes in circulating nutrient and hormone signals (including leptin, ghrelin and glucose) to impact feeding behaviour and energy expenditure. In line with this role the DMH shares reciprocal connections with the arc, VMH, LH and paraventricular nucleus (PVN) [28,29]. The DMH also sends primarily inhibitory GABAergic projections to sleep promoting areas including the VLPO, and excitatory glutamatergic connections to arousal promoting areas such as the LH and LC [30]. Galanin-expressing neurons in the DMH have been recently implicated in the balance of REM/NREM sleep states via distinct projections to the POA and raphe pallidus [31]. Importantly, the DMH is a prominent circadian centre in the mammalian brain; it is heavily innervated by SCN efferents via the subparaventricular zone [32], and is itself capable of maintaining autonomous clock function [33]. Consistent with this role, lesioning of the DMH in rats alters the distribution of REM and NREM sleep across the circadian cycle [34,35]. Clock gene expression and markers of neuronal activity in the DMH are also responsive to the timing of food intake, as highlighted in restricted feeding studies in laboratory rodents [36,37]. Thus, the DMH is a likely candidate for providing an anatomical relay to impose circadian timing and metabolic state-dependent responses onto classic sleep circuitry.

Alongside the LH and DMH, recent work has revealed the arcuate nucleus to be not only a major feeding centre, but also a potent site in the integration of feeding and sleep drives. Two populations of arcuate nucleus neurons control feeding behaviour and energy homeostasis; pro-opiomelanocortin (POMC) neurons are activated by feeding related factors such as insulin and leptin [38^•^,^39^] to inhibit food intake [40] and, in opposition, agouti-related protein (AgRP)/neuropeptide Y (NPY) expressing neurons are activated in response to food deprivation [41,42] and hunger signals such as ghrelin [43] to increase food seeking behaviours. In addition to their action on food intake, these orexigenic and anorexigenic neurons can have profound effects on sleep [44]. Golstein *et al.* recently showed that optogenetic and chemogenetic activation of AgRP neurons leads to increased wakefulness, and conversely that inhibition of AgRP neurons or activation of POMC neurons during starvation can rescue sleep behaviour at the expense of eating [45^••^]. Thus, the arcuate nucleus likely represents an important centre for coordinating regulation of sleep, wakefulness, and energy homeostasis.

The paraventricular thalamus (PVT), a structure, reciprocally connected to the SCN [46], has also been studied extensively with respect to its role in arousal and feeding behaviours [47,48]. Notably, the PVT receives dense innervation from NPY, MCH and orexin neurons [22,28], consistent with its role in the integration of energy balance. It was shown that starvation induced wakefulness increases activity in PVT neurons, specifically activating calretinin neurons [49^••^], while optogenetic activation of the PVT calretinin neurons induced wakefulness, while inhibiting them reduced starvation induced arousal [49^••^].

It is now clear that energy state imposes a profound influence on sleep regulation, and that integration of states of vigilance and energy state should be mediated by close interaction of neuronal populations within both sleep–wake and metabolic regulatory pathways. Prioritisation of arousal over sleep during times of negative energy balance has clear advantages for survival and this requires flexibility in sleep regulation. However, the question remains whether over long timescales, alterations in food availability can lead to more fundamental shifts in sleep amount, timing, and its homeostatic control.

## Sleep and circadian flexibility to shifting feeding schedules

Animals maintain a tight homeostatic control of sleep and wakefulness, which allows for a compensatory increase of sleep amount and intensity after extended wakefulness. Irrespective of the time of day, sleep deprivation invariably results in an increase of spectral EEG power in slow wave frequency range (so called slow-wave activity, SWA, 0.5–4 Hz) during NREM sleep, as well as increased sleep consolidation [50]. After the initial increase in SWA following sleep deprivation or spontaneous wakefulness, SWA then shows a progressive decline, and was therefore considered a sensitive measure of sleep need [11]. Under increased sleep pressure, such compensatory rebound of sleep SWA can manifest even during phases of the day when an individual is typically active [51^•^]. Conversely, excessive sleep during the time when animals are normally awake may lead to reduced sleep propensity during the subsequent inactive phase of 24 hour.

A useful paradigm to study the adaptive flexibility in sleep and metabolic homeostasis is through daytime restricted feeding (RF) in nocturnal laboratory rodents. It has long been known that animals will re-organise many behavioural and physiological processes in response to stable and recurrent feeding schedules [52]. Restricting food access to the light phase leads to an anticipatory rise in locomotor activity, body temperature and circulating corticosterone in advance of the new feeding time [53]. This daytime anticipatory behaviour is termed food anticipatory activity (FAA). The manifestation of FAA is independent of SCN control, with the SCN clock and rhythmic activity aligned to the prevailing light–dark (LD) cycle [54]. However, FAA exhibits properties of an underlying circadian clock process, and requires at least some of the canonical clockwork for its emergence [55,56]. Importantly, once established, behavioural entrainment of RF and development of FAA leads to a pronounced reorganisation of locomotor activity and sleep patterns across the day and night. It has been suggested that food anticipatory behaviour and the re-organisation of physiological and behavioural patterns during restricted feeding protocols are driven by one or more food entrainable oscillators (FEOs) [57]. However, the identity and location of such an FEO site and/or network have not been convincingly demonstrated.

**Figure 3:**
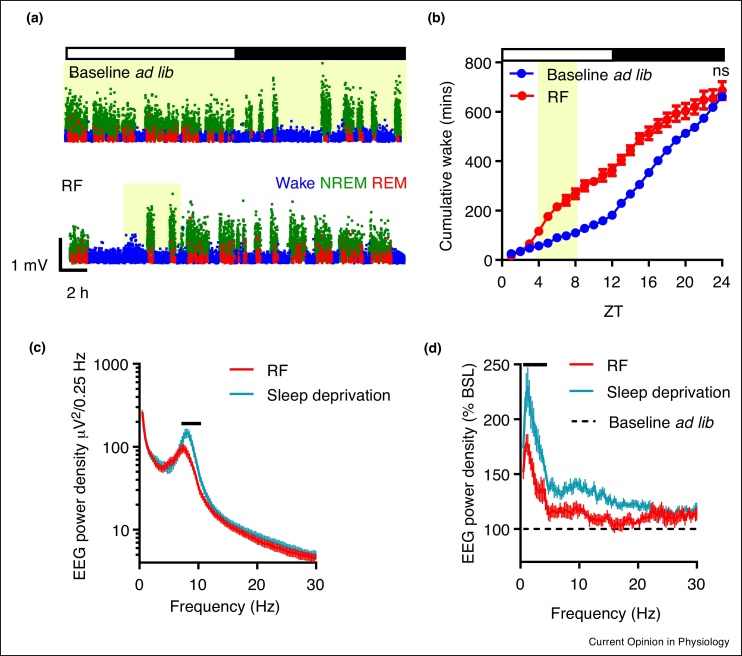
Sleep flexibility during entrainment to daytime restricted feeding in mice. **(a)** Daytime RF in mice results in profound disruption to typical sleep wake patterning **(b)** yet robust sleep homeostasis is still observed. **(c)** Wake is qualitatively different during RF in comparison to a similar duration of forced wakefulness under *ad lib* feeding conditions, and **(d)** SWA rebound is reduced in RF following this prolonged wakefulness. Yellow shading indicates food availability. Figure adapted from Ref. [61^••^].

In nocturnal rodents, entrainment to regular daytime feeding leads to gross changes in daily sleep–wake architecture, whereby daytime sleep lost due to FAA and feeding behaviours is compensated by increased sleep in the dark phase [58, 59, 60,61^••^]. We recently examined the impact of RF on sleep homeostasis in laboratory mice. Food availability was limited to a 4 hour window in the middle of the light period, with continuous monitoring of sleep and waking behaviours by EEG/EMG before and during 10 days of RF. We observed that during the first days after the commencement of the new feeding schedule, wakefulness was enhanced, yet the homeostatic response to sleep loss was diminished. However, once stable entrainment was achieved (based on the occurrence of robust FAA; from approx. day 7), the capacity to compensate for sleep loss was recovered. This manifested in the maintenance of the total daily amounts of wake and sleep that were equivalent during baseline (*ad libitum* feeding) and daytime RF (Figure 3), despite gross changes in their temporal organisation. Interestingly, our study also revealed that sleep intensity, as reflected in the levels of EEG SWA, was reduced during RF in comparison to baseline conditions or even following an imposed daytime 3 hour sleep deprivation. This change could be due to increased activity from wake-promoting subcortical areas in response to increased hunger cues, thus increasing cortical arousal [61^••^]. We surmise that when metabolic pressure is high, a decreased sleep drive allows animals to more readily maintain awake state and seek food at inappropriate times of 24 hour. This example of adaptive behavioural flexibility is consistent with the view that motivation-driven wakefulness may be less costly with regards to homeostatic accumulation of sleep debt [62,63] (Figure 1).

Similar to RF entrainment, mice will shift their typical nocturnal sleep–wake patterning to become relatively day-active when required to expend substantial energy to obtain food [64]. This shift to diurnal behaviour only occurs under negative energy balance, as working for palatable food reward (amid free access to normal chow) elicits increased treat-related work (wheel-running), yet does not induce a change in behavioural partitioning [65]. As discussed below, this shift in temporal niche is likely driven by a requirement to align energy saving states (such as daily torpor) into a specific circadian phase with appropriate reorganisation of other behaviours. These laboratory models offer a useful paradigm to explore sleep dynamics under increased foraging activity and energy conservation.

These studies indicate that during metabolic challenges such as food entrainment, sleep and wake can be re-organised drastically, both in the temporal domain, as well as with respect to their homeostatic control, which reveals profound flexibility in sleep regulatory mechanisms.

## Temporal partitioning of energy conservation, foraging and sleep under metabolic stress

Under conditions of food restriction and/or low ambient temperature, many small mammals (including mice) can engage daily torpor as a mechanism of energy conservation. Torpor is defined based on, primarily, a decrease in metabolic rates, as well as cessation of active behaviours and a depression of a broad range of physiological variables, including respiratory and heart rate [66,67]. Under chronic restricted feeding mice will exhibit daily bouts of torpor once stable entrainment to food has been established. The neural substrates regulating torpor remain elusive, but studies have suggested a role for hypothalamic and brainstem centres which control thermoregulation and energy homeostasis in both seasonal and fasting-induced torpor [68, 69, 70, 71]. Nutrient, endocrine, neuropeptide and monoamine responsive signalling pathways, which contribute to the manifestation of torpor (such as those involving glucose, leptin, ghrelin, adenosine, histamine, etc.), also exhibit significant overlap with both feeding and sleep circuitry [71, 72, 73, 74]. This should be of no surprise, as employment of such a pronounced yet highly controlled hypometabolic state requires substantial physiological and behavioural coordination, not least affecting the occurrence of sleep.

Both laboratory and field-based studies suggest that the timing of entry into and emergence from torpor is gated by the circadian clock, but also reflective of prevailing environmental conditions and internal energy state [68,75^••^,^76^]. The importance of circadian gating in torpor has been highlighted in more recent studies. For example, van der Vinne *et al.* used RF regimens in which frequency and timing of feeding differed from that imposed by the SCN clock (e.g. feeding cycles of 20 or 28 hour) in normal mice and those lacking clock function in the SCN [75^••^]. These studies suggested a dominance of circadian phase over previous feeding time in the emergence and timing of torpor [75^••^]. We recently used transgenic mice lacking *Gpr50*, which reliably enter stable torpor upon fasting, to explore the factors responsible for the regulation of torpor timing. When housed under constant lighting conditions (to remove external temporal cues), these mice exhibit two precisely timed bouts of torpor during a 48 hour fast [68]. Some caution must be applied to studies that employ long-term restricted feeding schedules which elicit circadian entrainment to timing of food availability (i.e. delivered at 24 hour intervals), as this will blur any distinction between adaption of the circadian system to food and any direct relationship between meal timing and torpor entry/emergence.

Circadian governance over torpor expression does not exclude a close link between torpor and sleep–wake regulation. Although torpor and sleep exhibit a number of physiological and behavioural similarities [77], electrophysiologically the state of torpor cannot be robustly classified as wake or sleep state, as hypothermia itself results in altered EEG patterns [78]. In addition, evidence suggests that torpor is associated with sleep deprivation-like effects in Djungarian hamsters (*Phodopus sungorus*), as post-emergence NREM sleep is characterised by increased EEG SWA [79,80], although EEG waves are not entirely typical for physiological sleep after sleep deprivation [81]. Specifically, early sleep under increased sleep pressure is typically characterised by steep slow wave slopes, indicative of greater cortical network synchronisation [82], while this is not observed during sleep immediately after emergence from torpor [81]. Similar post-torpor rebound sleep has been reported in the Gray Mouse Lemur (*Microcebus murinus*) [83].

It has been recently shown that galanin-expressing neurons of the POA regulate both sleep and body temperature. In mice, ablation of galanin POA neurons results in an elevated body temperature and an attenuated increase in EEG SWA after sleep deprivation [84^•^]. Interestingly, the same manipulation also prevented the occurrence of hypothermia after administration of a sedative drug dexmedetomidine. Although further research is needed to elucidate the underlying causes of these effects, the extensive overlap between neuronal circuities, which regulate states of vigilance and energy homeostasis, suggests that regulation of sleep and hypometabolic states is not independent. An interesting possibility is that entry into fasting-induced torpor is modulated by mechanisms involved in sleep promotion, which are, in turn, influenced by preceding sleep–wake history, metabolic state and the circadian clock. To this end, torpor represents an intriguing challenge to the prevailing views on sleep’s regulation and function, but also offers an opportunity for obtaining new insights into how the vital needs to eat and to sleep are balanced in the naturalistic environment.

## Conclusion

The need for sleep and feeding must be carefully balanced in order to maintain physiological homeostasis and enable survival. The circadian system provides a temporal framework both for sleep and wakefulness, as well as for the occurrence of torpor, which is employed as an energy saving strategy by many species. The mechanistic details of the circadian control of vigilance states and torpor under conditions of metabolic stress are yet to be fully elucidated. Recent work reveals a remarkable flexibility in sleep–wake timing, which can be regulated adaptively in response to metabolic demands and environmental pressures.

## Conflict of interest statement

Nothing declared

## Editorial disclosure

Given his role as Guest Editor, Vladyslav Vyazovskiy, had no involvement in the peer-review of this article and has no access to information regarding its peer-review. Full responsibility for the editorial process for this article was delegated to A. Jennifer Morton.

## References and recommended reading

Papers of particular interest, published within the period of review, have been highlighted as:

• of special interest
•• of outstanding interest
